# Effect of prenatal DHA supplementation on the infant epigenome: results from a randomized controlled trial

**DOI:** 10.1186/s13148-016-0281-7

**Published:** 2016-11-04

**Authors:** Susan J. van Dijk, Jing Zhou, Timothy J. Peters, Michael Buckley, Brodie Sutcliffe, Yalchin Oytam, Robert A. Gibson, Andrew McPhee, Lisa N. Yelland, Maria Makrides, Peter L. Molloy, Beverly S. Muhlhausler

**Affiliations:** 1 CSIRO Health and Biosecurity, PO Box 52, North Ryde, New South Wales 1670 Australia; 2 FOODplus Research Centre, School of Agriculture Food and Wine, The University of Adelaide, Adelaide, South Australia 5064 Australia; 3 CSIRO Data61, North Ryde, New South Wales 2113 Australia; 4 CSIRO Agriculture and Food, North Ryde, New South Wales 2113 Australia; 5 Child Nutrition Research Centre, South Australian Health and Medical Research Institute Adelaide, Adelaide, South Australia 5006 Australia; 6 Department of Neonatal Medicine, Women’s and Children’s Hospital, Adelaide, South Australia 5006 Australia; 7 School of Public Health, University of Adelaide, Adelaide, South Australia 5000 Australia

**Keywords:** Epigenetics, DNA methylation, Pregnancy, Fatty acids, Children, Randomized controlled trial

## Abstract

**Background:**

Evidence is accumulating that nutritional exposures in utero can influence health outcomes in later life. Animal studies and human epidemiological studies have implicated epigenetic modifications as playing a key role in this process, but there are limited data from large well-controlled human intervention trials.

This study utilized a large double-blind randomized placebo-controlled trial to test whether a defined nutritional exposure in utero, in this case docosahexaenoic acid (DHA), could alter the infant epigenome. Pregnant mothers consumed DHA-rich fish oil (800 mg DHA/day) or placebo supplements from 20 weeks’ gestation to delivery. Blood spots were collected from the children at birth (*n* = 991) and blood leukocytes at 5 years (*n* = 667). Global DNA methylation was measured in all samples, and Illumina HumanMethylation450K BeadChip arrays were used for genome-wide methylation profiling in a subset of 369 children at birth and 65 children at 5 years.

**Results:**

There were no differences in global DNA methylation levels between the DHA and control group either at birth or at 5 years, but we identified 21 differentially methylated regions (DMRs) at birth, showing small DNA methylation differences (<5%) between the treatment groups, some of which seemed to persist until 5 years. The number of DMRs at birth was greater in males (127 DMRs) and in females (72 DMRs) separately, indicating a gender-specific effect.

**Conclusion:**

Maternal DHA supplementation during the second half of pregnancy had small effects on DNA methylation of infants. While the potential functional significance of these changes remains to be determined, these findings further support the role of epigenetic modifications in developmental programming in humans and point the way for future studies.

**Trial registration:**

Australian New Zealand Clinical Trials Registry (ANZCTR), ACTRN12605000569606 and ACTRN12611001127998

**Electronic supplementary material:**

The online version of this article (doi:10.1186/s13148-016-0281-7) contains supplementary material, which is available to authorized users.

## Background

Human and animal studies have provided clear evidence that the environment during critical periods of development can increase the risk of a wide range of diseases in postnatal life [1, 2]. This programming of disease risk may be mediated, at least in part, by (semi) permanent epigenetic alterations which can regulate gene expression and thereby affect phenotype. In animal studies, controlled nutritional interventions before or during pregnancy have been shown to significantly affect the epigenome and to be associated health outcomes [3, 4]. In humans, epidemiological studies have provided evidence that different intrauterine exposures, including malnutrition [5, 6], smoking [7], toxicants [8] and micronutrient deficiencies [9], can affect the epigenome of the infant. A limited number of small clinical trials involving specific prenatal interventions (i.e. micronutrient supplementation [10, 11] and weight loss surgery [12]) have produced similar findings; however, large, well-controlled trials in humans are required to confirm the ability of defined nutrition in pregnancy to impact on the infant epigenome.

Fish oil supplements which contain the long chain n-3 polyunsaturated fatty acids (n-3 LCPUFAs) eicosapentaenoic acid (EPA) and docosahexaenoic acid (DHA) are commonly consumed during pregnancy. These fatty acids have received attention for their health benefits, specifically in relation to diseases with an inflammatory component [13, 14]. DHA also plays an important role in the development of the brain and central nervous system [15], and exposure to an increased DHA supply in utero has been associated, in some studies, with a reduced risk of allergy and improved metabolic health outcomes in postnatal life [16, 17]. The mechanisms underlying these effects are unclear; however, there have been suggestions that they may be epigenetically mediated.

In rats, intake of n-3 LCPUFAs during pregnancy was shown to change DNA methylation levels in the fatty acid desaturase promoter in the liver of the offspring [18]. In other studies, an effect of DHA on methylation in the placenta via the alteration of one carbon metabolism has been proposed [19, 20]. In adult humans, differences in DNA methylation have been identified between people with high and low n-3 PUFA intakes [21, 22], and n-3 PUFA supplementation has been shown to induce changes in methylation at specific CpG sites [23].

There are currently only two studies which have investigated the effects of increased prenatal DHA exposure on DNA methylation in the neonate. One trial in 260 subjects used a candidate gene approach and showed that maternal DHA supplementation induced small changes in global DNA methylation and methylation at IGF2/H19 imprinted genes in cord blood [24, 25], while another trial in 70 subjects assessed genome-wide DNA methylation in CD4+ T cells from cord blood and did not find any substantial effect of prenatal DHA supplementation [26]. Thus, additional genome-wide methylation studies with large sample sizes are needed to determine the impact of prenatal DHA supplementation on the epigenome of the infant, and whether this could underlie the effects of maternal DHA supplementation on infant and child outcomes.

The aim of this study was to utilize DNA samples obtained from a large randomized controlled trial, DOMInO (*D*HA to *O*ptimise *M*other *In*fant *O*utcome), to determine the impact of a defined nutritional intervention, specifically a high-dose DHA supplement, during the second half of pregnancy on the epigenome of the children at birth and at 5 years of age.

## Results

### Subject characteristics

Characteristics of the mothers and children included in the study are summarized in Table 1. There were no significant differences in the key characteristics between the DHA and the control group pre-randomization, either in the whole study population or in any of the subsets used for genome-wide methylation analysis. As expected, there were significant differences in cord blood DHA concentrations between the groups post randomization.

**Table 1:** Characteristics of mothers and children in the control and DHA-supplemented groups

	Newborn subset for 450K BeadChip (*n =* 369)		All newborns (*n =* 991)	
	DHA group (*n =* 190)	Control group (*n =* 179)	DHA group (*n =* 517)	Control group (*n =* 474)
Recruitment hospital				
Flinders Medical Centre and private hospitals, no. (%)	59 (31.1)	64 (35.8)	199 (38.5)	188 (39.7)
Women’s and Children’s Hospital, no. (%)	131 (68.9)	115 (64.2)	318 (61.5)	286 (60.3)
Primiparous, no. (%)	68 (35.8)	60 (33.5)	212 (41.0)	192 (40.5)
Mother’s age at trial entry, mean (SD), years	30.2 (5.9)	30.2 (5.5)	29.8 (5.6)	29.7 (5.5)
Mother smoking at trial entry, no. (%)	19 (10.0)	15 (8.4)	60 (11.6)	52 (11.0)
Duration of gestation, median (IQR), weeks	39.9 (38.6–40.7)	39.7 (39–40.5)	39.7 (38.7–40.7)	39.6 (38.9–40.4)
Birth weight, median (IQR), g	3542 (3160–3859)	3510 (3260–3832)	3500 (3160–3820)	3510 (3190–3798)
Child male sex, no. (%)	92 (48.4)	93 (52.0)	260 (50.3)	242 (51.1)
DHA in cord blood, mean (SD), % of total fatty acids	7.9 (1.8)*	6.4 (1.5)	7.8 (1.8)*	6.3 (1.5)

*Abbreviations*: *DHA* docosahexaoneic acid, *IQR* interquartile range

*Difference between the DHA and control group *P* < 0.001

### Global DNA methylation

No significant differences in LINE1 hypomethylation levels were found between the control and DHA-supplemented groups either at birth or at 5 years of age (Table 2). Furthermore, no association was found between DHA concentration in cord blood and LINE1 DNA methylation in the whole study group, or when separated by treatment group or gender (data not shown). Consistently lower LINE1 hypomethylation levels were found in males compared to females at both time points (*P* < 0.001) and both in males and females mean LINE1 hypomethylation levels were lower at 5 years compared to birth (*P* < 0.001), independent of treatment group.

**Table 2:** LINE1 hypomethylation at birth and at 5 years for the DHA and control group

Subgroup	DHA group mean (SD)	Control group mean (SD)	Treatment effect DHA-control adjusted^a^ (95% CI)	*P* value, adjusted^a^
Birth	1.47 (0.57)^b^	1.43 (0.45)	0.04 (−0.02, 0.10)	0.211
5 years	0.99 (0.15)	0.99 (0.16)	0.00 (−0.02, 0.03)	0.706
Birth, female	1.56 (0.64)	1.50 (0.48)	0.06 (−0.04, 0.15)	0.229
Birth, male	1.39 (0.49)	1.36 (0.42)	0.03 (−0.06, 0.11)	0.583
5 years, female	1.04 (0.16)	1.03 (0.17)	0.01 (−0.03, 0.04)	0.655
5 years, male	0.95 (0.13)	0.95 (0.15)	0.00 (−0.03, 0.03)	0.837

All children who had any global DNA methylation data were used for this analysis; 522 children had data at birth and 5 years, 469 had data at birth only, 141 had data at 5 years only

^a^Adjustment was made for centre, parity and gender

^b^LINE1 methylation values are expressed as relative hypomethylation values compared to a reference sample

At birth no significant differences between the treatment groups were found in mean DNA methylation levels by annotation across all probes on the 450K array (Additional file 1: Table S1). Small DNA methylation differences between males and females were found across all designations, with males showing higher DNA methylation levels than females. These results were similar at age 5 years (data not shown) and consistent with the lower hypomethylation levels of LINE1 repeats observed in males compared to females.

### Genome-wide DNA methylation

#### Variable methylated sites and regions

CpG sites that display high inter-individual variation in DNA methylation levels are considered to be most susceptible to environmental effects, including nutrition [27]. In our study population, we first identified the most variable sites and variable methylated regions (VMRs) in the whole population, irrespective of treatment group. In total, 0.4% of the probes on the arrays showed a variance in methylation beta values of >0.01 across the study population at birth and 0.6% of the probes showed a variance of >0.01 across the study population at age 5 years.

In the study population at birth, 5296 VMRs were identified, and 4214 VMRs were identified at 5 years of age. Of these VMRs, 3135 showed either complete or partial overlap across the two time points (Additional file 1: Table S2).

**Fig. 1:**
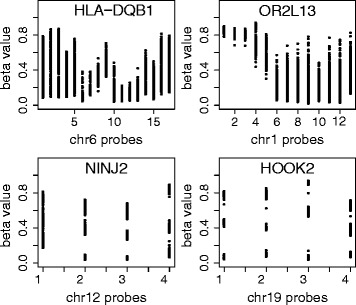
Variable methylated regions (VMRs) at birth. Every *dot* represents the DNA methylation level for a child at a single probe within the VMR

Multiple highly significant VMRs were located in probe dense, polymorphic genomic regions such as the Major Histocompatibility Complex (MHC) region on chromosome 6 (*HLA-DQB1* and *HLA-DRB1*) and in the olfactory receptor gene *OR2L1*3. Visualization of individual methylation beta values (Fig. 1) for VMRs revealed that while many CpGs within a VMR showed an even distribution of beta values across samples, for some CpGs within VMRs, such as for *HOOK2* and *NINJ2* (Fig. 1), the samples clustered into two or three distinct groups, indicating that these VMRs were likely due to genetic variation. Thus, children that were homozygous for a particular variant displayed either low or high methylation levels while heterozygous children displayed an intermediate level of methylation, often for multiple consecutive CpG sites. Such genetic influence on levels of DNA methylation may extend in cis across hundreds of kilobases [28, 29], and genetic and environmental variation may further interact to determine DNA methylation levels [30].

#### Effect of prenatal DHA supplementation on genome-wide DNA methylation

We first analysed the DNA methylation data at an individual probe level and found no differentially methylated probes between the DHA and control groups at birth at an FDR-adjusted *P* value of <0.05. When analyses were undertaken separately in males and females, no differentially methylated probes were found in males, but in females, one probe (cg00870514, near the transcription start site of *RAB11FIP4*) showed significantly higher DNA methylation levels in the DHA compared to the control group (0.54 ± 0.03 vs 0.51 ± 0.03, adjusted *P* value =0.004). A comparison of the *limma P* value and *t*-statistic distributions for the treatment group analyses in males, females and the whole population showed that, overall, the *P* values in males were slightly lower than in females or the combined population (Additional file 2: Figure S1).

Methylation at neighbouring CpG sites is often correlated, so extra analyses were done to identify differentially methylated regions (DMRs); genomic regions of multiple consecutive probes showed differential methylation between the treatment groups. This regional grouping of probes provides greater power to identify methylation differences that may be associated with functional elements. The DMR analysis in the combined population identified 21 DMRs between the treatment groups at birth (Table 3). Adjustment for cell mixture did not significantly change the main study outcomes; DMRs identified in unadjusted data are presented in Table 3 and adjusted data in Additional file 1: Table S3).

**Table 3:** Differentially methylated regions between the DHA-supplemented and control group at birth

	hg19 coordinates	No. of probes	Size (bp)	Gene	Genomic location	Min *P* value^a^	Max beta diff (DHA-control)^b^	VMR overlap
1	chr3:138152837-138153763	12	926	ESYT3	TSS1500,TSS200,1stExon,5′UTR	5.75E−15	−0.045	Y
2	chr12:132293329-132293702	5	373		Intergenic	1.12E−07	−0.026	Y
3	chr15:34610829-34611069	4	240	SLC12A6	Body,1stExon,5′UTR,TSS200	4.99E−06	−0.011	Y
4	chr3:42306150-42307193	10	1043	CCK	5′UTR,1stExon,TSS200,TSS1500	6.05E−06	−0.012	Y
5	chr7:1882776-1883876	9	1100	MAD1L1	Body	8.75E−06	0.023	Y
6	chr2:48844728−48845068	8	340	GTF2A1L, STON1	TSS1500,Body,TSS200,1stExon,5′UTR	8.75E−06	−0.019	Y
7	chr6:166876490-166877038	7	548	RPS6KA2	Body	8.75E−06	0.022	Y
8	chr8:59058254-59058585	3	331	FAM110B	5′UTR	9.79E−06	−0.009	N
9	chr10:134221633-134222453	6	820	PWWP2B	3′UTR,Body	1.10E−05	−0.033	Y
10	chr3:42201314-42201898	6	584	TRAK1	TSS1500,Body,TSS200,1stExon	2.11E−05	−0.015	N
11	chr6:150346721-150347053	10	332	RAET1L	TSS200,TSS1500	5.41E−05	−0.009	Y
12	chr12:7781004-7781431	5	427		Intergenic	2.15E−04	−0.044	Y
13	chr6:1619162-1619687	3	525		Intergenic	2.62E−04	−0.012	N
14	chr8:117950244-117950504	7	260	C8orf85	TSS1500,TSS200,1stExon,5′UTR	4.08E−04	−0.01	N
15	chr4:62382932-62383240	4	308	LPHN3	Body	6.20E−04	−0.019	N
16	chr4:1107202-1107259	2	57	RNF212	1stExon,5′UTR	6.45E−04	−0.01	N
17	chr17:75446431-75446661	6	230	SEPT9	TSS200,Body,1stExon,5′UTR	7.94E−04	−0.013	N
18	chr6:31549929-31550090	2	161	LTB	Body,1stExon	8.92E−04	−0.008	N
19	chr22:32599511-32599516	2	5	RFPL2	TSS200,5′UTR,TSS1500	9.17E−04	−0.037	Y
20	chr20:13620031-13620048	2	17	TASP1	TSS1500	9.61E−04	0.012	N
21	chr1:156261200-156261207	2	7	TMEM79	Body	9.77E−04	0.01	N

*Abbreviations*: *DMR* differentially methylated region, *VMR* variable methylated region, *TSS1500* 1500 base pairs from the transcription start site, *TSS200* 200 base pairs from the transcription start site, *5′UTR* 5′ untranslated region, *3′UTR* 3′ untranslated region

^a^Smallest *P* value for a probe in the DMR

^b^Largest DNA methylation (beta value) difference between the DHA and control group for a probe within the DMR

Of the 21 identified DMRs, 18 DMRs were associated with a gene and 3 DMRs were intergenic. We then determined the overlap of DMRs with VMRs and found that about half of the DMRs were either completely overlapping or contained within a VMR, which supports the prevailing hypothesis that highly variable regions are most susceptible to alteration by the (prenatal) environment [27, 31].

**Fig. 2:**
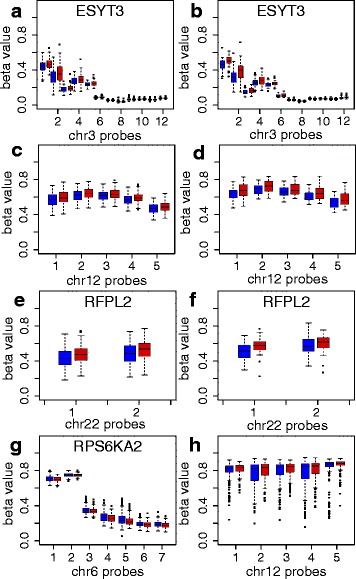
Differentially methylated regions (DMRs) between the DHA and control group. DMRs identified at birth (**a**, **c**, **e**, **g**, **h**) and the corresponding region at age 5 years (**b**, **d**, **f**). The DHA group is shown in *blue*, and the control group in *red*. The *bottom* and *top* of the box represent the 25th and 75th percentile of DNA methylation within the group, respectively, and the middle band the median DNA methylation value

Overall, methylation differences between the DHA and control groups were modest, with maximum group beta differences of 4.5% for single probes within DMRs. The differences between the DHA and control groups were, however, consistent over multiple consecutive probes within each DMR (Fig. 2). The majority of the DMRs (17/21) showed lower methylation levels in the DHA group compared to the control group.

The DMRs identified at birth were located in genes with a variety of functions, including lipid exchange between membranes (*ESYT3* [32]), plasma membrane function (*SLC12A6* [33]), appetite regulation (*CCK* [34]), immune function (*RAET1L* and *LTB* [35, 36]) and neurodevelopment/brain function (*SLC12A6*, *TRAK1*, *LPHN3* and *RFPL2* [33, 37, 38]).

We further analysed males and females separately. When separated by gender, a greater number of DMRs were identified both in males (127 DMRs) and females (72 DMRs) (Additional file 1: Tables S4 and S5). Three of these DMRs were identified in both males and females (in *ESYT3*, *TNXB* and a region in chr 7) of which one DMR (in *ESYT3*) showed methylation differences between treatment groups in the same direction in males and females.

For the identification of the DMRs, we had relaxed the default parameterization of DMRcate, which may have increased the number of false positive DMRs. We applied permutation testing to determine the likelihood that identified DMRs arose by chance. For the combined as well as gender-specific analyses data, the number of DMRs—combined data set (*n =* 21), males (*n =* 127), females (*n =* 72)—were higher than the median number of DMRs that would be expected by chance alone (*n =* 10 for the combined set, *n =* 12 for males, *n =* 13 for females and, Additional file 2: Figure S2). This suggests that most of the identified DNA methylation differences are true positive findings. Particularly for the gender-specific DMRs, the likelihood of the numbers of DMRs observed occurring by chance is less than 1 or 2%.

**Fig. 3:**
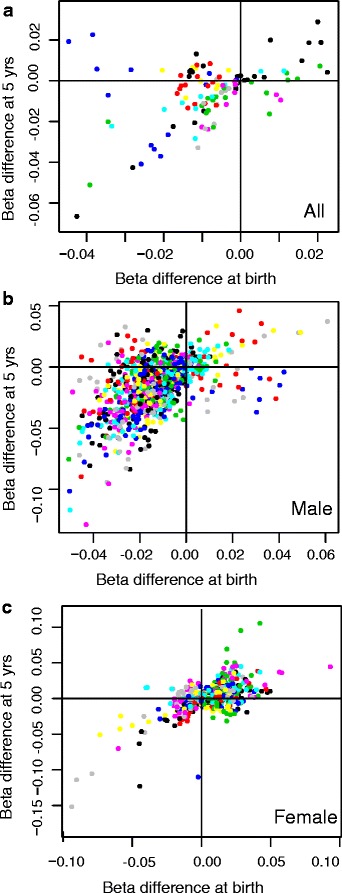
DNA methylation differences between treatment groups for probes within significant differentially methylated regions (DMRs). The differences in beta values between the DHA and the control group at birth and age 5 years are shown for probes that are part of significant DMRs between the DHA and control group at birth. DNA methylation differences are shown for all children (**a**) and males (**b**) and females (**c**) separately. The probes are coloured by DMR; DMR1 (ESYT3) in *black* in the *lower left corner*, DMR2 (chr12) in *blue* in the *lower left corner*, DMR12 (chr12) in *blue* in the *upper left corner*

At 5 years of age, 10 DMRs, but no significant probes, were identified between the DHA and the control groups (Additional file 1: Table S6). All these DMRs showed lower methylation levels in the DHA group compared to the control group, consistent with the findings at birth. The DMRs found at 5 years were all different from the DMRs at birth. However, a comparison of DNA methylation differences between treatment groups at birth and at 5 years revealed that the methylation changes for probes within the 21 DMRs identified at birth, although not reaching genome-wide statistical significance at age 5 years, were generally in the same direction at age 5 years (Fig. 3). In the case of the top most significant DMRs at birth (in *ESYT3* and at chr12), the magnitude of the group differences in DNA methylation at the two time points were very similar (Figs. 2 and 3). For other DMRs, such as another DMR on chr 12 (DMR12 in Table 3 and in Fig. 3), DNA methylation differences were present at birth, but no longer present at age 5 years.

When comparisons between treatment groups at age 5 years were conducted separately in males and females, 45 DMRs were found in males and 10 DMRs were found in females (Additional file 1: Tables S7 and S8). Six DMRs in males were identified both at birth and at 5 years (Additional file 1: Tables S4 and S7), with lower DNA methylation levels in the DHA compared to the control group at both time points. Comparison of the DNA methylation differences between groups in males and females at both time points showed that, similar to what was observed in the combined population, there was a trend for maintenance of the differences between treatment groups over time (Fig. 3).

### Association of DNA methylation with DHA concentration at birth

Previous analyses have shown that while the children in the DHA group on average had higher DHA concentrations in cord blood than the control group, there was substantial overlap in DHA levels between the groups. Therefore, in addition to comparing DNA methylation between the treatment groups, we also investigated the association between DNA methylation and the DHA concentrations measured in cord blood. Methylation levels for 349 probes (FDR *P* value <0.05) were significantly associated with DHA concentration. Because of the known association of DHA concentration with gestational age, gestational age was added as a covariate to the model, and no probes remained significantly different between groups after this adjustment. In addition, no probes were found to be significantly associated with cord blood DHA concentration when the analyses were performed separately by group or sex.

## Discussion

In this large, well-characterized study population, we showed that maternal DHA supplementation across the second half of pregnancy was associated with modest alterations in DNA methylation in a small subset of genomic regions within genes involved in diverse biological processes. The strengths of our study include the randomized design, the large sample size and the DNA methylation analysis at two time points, which enabled us to determine whether any DNA methylation changes induced by DHA persisted over time.

To the best of our knowledge, there are currently only two other, smaller, randomized controlled trials that have investigated the effects of prenatal DHA supplementation on the epigenome at birth [24–26], and our study is the first to determine whether any epigenetic influences of DHA persist in childhood. In line with our findings, these previous studies reported minor or no effects of prenatal DHA supplementation on DNA methylation in either cord blood [24, 25] or infant CD4+ T cells [26]. In the study by Lee et al., a small effect of DHA supplementation on global DNA methylation was reported in children from mothers who smoked during pregnancy, while an effect on *IGF2* promoter methylation was identified only in preterm infants [24, 25]. This suggests that prenatal DHA exposure may have a greater impact on the epigenome in some subgroups. We did not find specific effects of DHA on the epigenome in subpopulations of preterm infants or in children from smokers (data not shown), but our study may have been underpowered to detect differences in these small subgroups.

The study by Amarasekera et al. included 70 mother-child pairs, and although a high dose of DHA was consumed during pregnancy (~2 g/day), no significant effect on DNA methylation was observed [26]. In agreement with our results, there were no probes associated with cord blood DHA concentrations in Amarasekera’s study. However, it is difficult to separate effects of cord blood DHA concentration on DNA methylation from those of gestational age. Both DNA methylation profiles and cord blood DHA have been reported to be related to gestational length [39]. Importantly, in the DOMInO trial, DHA supplementation resulted in a significant increase in the length of gestation and cord blood DHA concentrations were positively correlated with gestational age [40]. Further studies are needed to assess the independent contribution of these variables to DNA methylation profiles.

The effects of DHA supplementation on DNA methylation in our study showed a clear gender effect and appeared to be more pronounced in males compared to females, suggesting that the epigenome of males may be more susceptible to prenatal exposure to DHA. This is in line with the results of a number of previous studies, which have reported sex differences in response to the prenatal environment, with most identifying more pronounced effects in males [41–43]. Differences in placental adaptations to changes in the prenatal environment have been suggested as a mechanism underlying these sex-specific effects [41, 43]. Widespread sex differences in DNA methylation, independent of DHA, may also have influenced the response.

We saw limited overlap between the significant DMRs at birth and at age 5 years in our study population. However, DNA methylation differences between treatment groups for a number of the DMRs identified at birth, while not statistically significant, were still present at age 5 years. Given the small size of the DNA methylation differences between treatment groups relative to their within-group variance, it is possible that there was insufficient statistical power in the smaller subset of children assessed at age 5 years for these differences to reach genome-wide statistical significance. Our results do, however, raise the possibility that small DNA methylation changes induced by increased DHA exposure may persist over time, at least to some extent. Genetic variation between treatment and control groups, alone or interacting with DHA supplementation [30, 44], could contribute to the observation of persistence over time for some DMRs. However, the similarity of effect in the birth comparison and the subset of subjects assessed at 5 years suggests the effects are predominantly due to treatment. Larger studies assessing multiple time points in childhood are needed to confirm this.

For many DMRs, the initial methylation differences between groups were no longer present at 5 years of age. This is not unexpected, since the maternal environment may still have a proportionally high impact on DNA methylation in the immediate postnatal period in comparison to later childhood, at which time the number of postnatal exposures with the potential to modify the epigenome accumulate and the effect of prenatal exposures is diluted. In support of this, studies investigating the impact of maternal smoking on the child’s epigenome have demonstrated that only part of the smoking-induced epigenetic changes persisted into adolescence [45].

Overall, the data from both our study and the two previous DHA intervention trials do not support the existence of a strong effect of DHA supplementation during the second half of pregnancy on DNA methylation in blood cells of infants. This could be due to a variety of reasons. First, the DNA methylation profile in blood may not necessarily reflect the profile of target tissues, particularly those which contain relatively high proportions of DHA such as the brain or adipose tissue. An alternate possibility is that DHA may have had effects on the epigenome via mechanisms other than DNA methylation, such as histone modifications, that were not investigated in our study. The natural inter-individual variation in blood cell populations may also have introduced variation in DNA methylation, thereby masking effects of the intervention. However, this possibility seems unlikely since the outcomes of our study were very similar when the analyses were adjusted for cell mixture [46]. A study using purified CD4+ T cells also found no substantial effects of prenatal DHA supplementation on DNA methylation in the newborn [26].

The modest effects of maternal DHA supplementation are consistent to some extent with the findings of the DOMInO trial and its associated follow-up studies, which have reported modest or no effect of the maternal DHA supplementation on growth, neurodevelopment and fat mass [40, 47, 48]. Positive effects of DHA were noted for immune-related outcomes at age 1 year in a subset of the DOMInO children at high hereditary risk of allergic disease [49]. This suggests that specific subgroups of children benefit more from the intervention and may also show more pronounced changes in DNA methylation. The timing of the DHA supplementation may have limited the impact on the epigenome, since the major period of epigenetic remodelling of the fetus is early in pregnancy, and the intervention did not begin until ~20 weeks gestation in the DOMInO trial. While there are major practical and ethical complications involved in testing the effects of interventions applied earlier in pregnancy, studies on naturally occurring changes of food availability due to seasonal differences in rural Gambia or supply restriction in the Dutch Hunger Winter have provided evidence that exposures early in pregnancy generally have a more profound effect on the epigenome compared to exposures later in gestation [5, 6].

Overall, while the observed changes in DNA methylation in our study were relatively small, small changes in DNA methylation in regulatory parts of the genome, such as in gene promoter or enhancers, can nevertheless associate with significant effects on gene regulation. Whether the DNA methylation differences observed in our study translate into functional consequences for gene expression and phenotype remains to be determined.

## Conclusions

We demonstrated, using a large, randomized controlled trial, that maternal DHA supplementation across the second half of pregnancy had modest effects on DNA methylation at specific regions of the genome in blood cells of children at birth, some of which seem to persist until 5 years of age. We also observed sex-specific effects of the intervention on DNA methylation, and in general, effects appeared to be more pronounced in males than females. There is still relatively little human data on the magnitude and extent of epigenetic changes impacted by nutrition during pregnancy. Our results adds to the growing body of evidence implicating epigenetic regulation as a mechanism underlying the impact of nutritional exposures in utero on infant and child health in humans, but also points to the need for similar studies earlier in pregnancy.

## Methods

### Study population

The present study is part of the EpiSCOPE Consortium [50] and uses biological samples collected within the first few days after birth (neonatal samples) and at 5 years of age, as part of the growth and insulin resistance follow-up of children whose mothers participated in a registered, multi-centre, double-blind randomized controlled trial, the DOMInO trial (ACTRN12605000569606 and ACTRN12611001127998). Details of the design of the DOMInO trial and the follow-up study have been previously published [40, 48]. Briefly, women less than 21 weeks’ gestation were randomly allocated to consume three capsules a day providing either ~800 mg/day DHA and ~100 mg/day EPA (Incromega 500 TG, Croda Chemicals, East Yorkshire, UK) or a similar dose of vegetable oil without DHA, until delivery. The randomization was performed using a computer-driven telephone randomization service with stratification by enrolling centre and parity (first or subsequent birth). Adherence to the intervention was monitored, and DHA concentrations were measured in plasma phospholipids from cord blood according to previously reported methods [40].

DOMInO participants who had been enrolled at one of the Adelaide centres (Women’s and Children’s Hospital or Flinders Medical Centre; *n =* 1660) and had not withdrawn or died were contacted when their child was 2.5 years of age and invited to participate in the growth and insulin resistance follow-up of the DOMInO children at 3 and 5 years of age. Those who provided written informed consent (*n =* 1531) were enrolled in the follow-up study, and their child was invited to attend clinic appointments at 3 and 5 years of age. At the 5-year appointments, between 25 March 2009 and 4 October 2013, a blood sample was collected from those children whose primary carer consented to this procedure. The blood samples were collected into EDTA blood tubes and placed on ice; most samples were processed within 4 h and all samples within 24 h of collection. DNA was isolated from the buffy coat of the samples (*n =* 667). For the neonatal samples, written informed consent was sought from all participants involved in the growth and insulin resistance follow-up study to access their child’s newborn screening card for the purposes of isolating DNA for (epi)genetic studies. All procedures were conducted in accordance with the trial protocol and approved by the local institutional ethics committees.

### Sample collection and DNA extraction

Neonatal screening (Guthrie) cards were retrieved from a dedicated storage facility, in which the cards had been stored at room temperature for between 5 and 7 years prior to use. Blood spot punches were taken from Guthrie cards of 991 DOMInO children whose mothers had given consent. DNA was extracted using GenSolve technology (IntegenX, Pleasanton, CA, USA) followed by purification using the QIAamp DNA micro kit (Qiagen, Doncaster, VIC, Australia) and an additional ethanol precipitation step. Briefly, three punches from a Guthrie card, each 3 mm in diameter, were incubated for 1 h at 65 °C in 620 μL recovery solution A in 1% lithium dodecyl sulfate, in the presence of protease. After incubation, the blood spots were transferred to a spin basket in a new tube and centrifuged for 2 min at full speed for optimal recovery of the lysate. Subsequently, the spin basket and blood spots were discarded and 20 μL of recovery solution B was mixed with the lysate before proceeding with the DNA purification. A volume of 600 μL ethanol was added to the lysate and loaded onto a QIAamp micro column. The DNA was purified on the column according to the manufacturer’s instructions with the modification that the columns and the elution buffer were incubated for 10 min at 70 °C prior to the final centrifugation step. After elution, the DNA was ethanol precipitated with 30 μg Glycoblue as a carrier (Life Technologies, Mulgrave, VIC, Australia) and the resulting pellet was dissolved in 60 μL AE buffer with 0.01% Triton X100.

DNA from the peripheral blood leukocyte samples collected at age 5 years was extracted using the QIAquick DNA extraction kit (Qiagen, Doncaster, VIC Australia) as per the manufacturer’s instructions. The quality and quantity of all DNA samples was assessed using a Nanodrop spectrophotometer and the Quant-iT Picogreen dsDNA assay (Life Technologies, Mulgrave, VIC, Australia).

### DNA methylation

#### Global DNA methylation

Hypomethylation levels in long interspersed nuclear elements-1 (LINE-1) in all newborn (*n =* 991) and 5-year (*n =* 663) DNA samples were quantified using the end-specific PCR assay [51]. In this assay, the relative cutting of the DNA by the methylation-sensitive enzyme HpaII (GeneSearch, Arundel, QLD, Australia) is compared with that of the methylation-insensitive enzyme Dra1 (GeneSearch, Arundel, QLD, Australia) to give a measure of DNA hypomethylation. The method depends upon the use of 5′-tailed, 3′-blocked oligonucleotides called facilitator oligonucleotides (Foligos). Only cut DNAs with specific matching sequences at their 3′ ends can copy the tails of the Foligos and thus become tagged and available for subsequent PCR. Samples were run in triplicate and the LINE1 hypomethylation levels in the DNA samples were normalized relative to an external reference DNA sample from human blood (Roche Applied Sciences, Dee Why, NSW, Australia).

The effect of DHA supplementation on global methylation was analysed using a linear regression model with a generalised estimating equation (GEE) to account for repeated measures. A treatment-by-time interaction term was included in the model and estimates of treatment effect were derived separately for birth and 5 years. Both unadjusted and adjusted analyses were performed, with adjusted analyses including centre, parity and sex as covariates. The possibility of effect modification by infant sex was investigated by fitting separate models for birth and 5 years, with a treatment-by-sex interaction term included in the model; separate estimates of treatment effect were derived for males and females. Differences in global DNA methylation according to sex and time, irrespective of the treatment group, were investigated by fitting a separate sex-by-time model with GEE to account for repeated measures.

### Genome-wide DNA methylation analysis

**Fig. 4:**
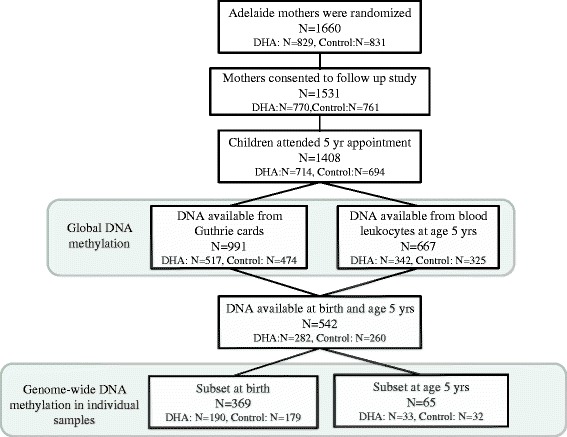
Flow diagram of the number of children included in the study

Genome-wide, site-specific DNA methylation was assessed using the Illumina Infinium HumanMethylation450 BeadChip (450K) in samples from a subset of 369 children at birth and a subset of 65 children at age 5 years (Fig. 4). The subset at birth comprised those children for which a sufficient quantity of DNA was obtained from the neonatal screening card (>250 ng) and for whom DNA samples were also available at age 5 years. The subset at age 5 was randomly selected from the full data set by an independent statistician to contain an equivalent number of males and females and an equivalent number from each treatment group.

All selected DNA samples were submitted to the Australian Genome Research Facility (Parkville, VIC, Australia) for bisulfite conversion using the Zymo EZ DNA Methylation kit (Zymo Research, Irvine, CA). Bisulfite-treated DNA was hybridized to the 450K arrays and the arrays were processed following standard protocols. Samples were randomized across arrays according to treatment group, sex and date of birth.

Normalization of the raw intensity data was performed using the *dasen* method from the Bioconductor software package wateRmelon [52]. The union of probes located on the sex chromosomes (*n =* 11,648), probes targeting CpGs located two or fewer nucleotides from a known single nucleotide polymorphism (SNP) with a minor allele frequency >0.05 (*n =* 29,476), and known cross-hybridizing probes (*n =* 30,969) [53] were excluded from the analysis. Probes were also excluded if they failed in one or more samples, based on a detection *P* value >0.05.

Principal components analysis showed that batch effects were present across the slides (groups of 12 arrays) for the arrays at birth, but not at age 5 years. These batch effects for the arrays at birth were removed using the Harman software package [54]. This method computes, and removes as noise, batch-to-batch variability in the data to the extent that it cannot be accounted for by the observed biological variance with an acceptable probability. The batch noise was removed with the trade-off coefficient set at 95% in favour of preserving (biological) signal, i.e. the probability of losing genuine signal in the process of removing noise was kept at 0.05. Newborn data after batch correction was used in all analyses, except for the analysis of variably methylated regions (VMRs) that showed SNP-associated effects.

The genome-wide DNA methylation data was first used to give an estimate of the global DNA methylation level at birth. The mean beta value for all probes on the array was calculated as well as the mean beta for probes based on their genomic annotation according to the Illumina 450K manifest file. A Welch unequal variance *t* test was used for comparison of global methylation levels between the treatment groups and sexes.

To identify differentially methylated probes in the DHA group compared to the control group, the limma package [55] was used on quantile normalized beta values to compute a moderated *t* test. Analyses were performed with adjustment for the stratification variables parity and centre of birth, as well as sex. The analyses were also performed with and without an adjustment for cell mixture using reference data on cell-specific DNA methylation signatures for cord blood [46]. Since adjustment for cell mixture did not significantly change the main study outcomes (Additional file 1: Table S3), only uncorrected data is presented in the main paper. *P* values were corrected for multiple testing using the Benjamini-Hochberg method [56] and significant differentially methylated probes were identified based on a false discovery rate (FDR) *P* value <0.05.

Testing for VMRs, independent of treatment group, and for differently methylated regions (DMRs) between the treatment groups was carried out using the Bioconductor package DMRcate [57], which extracts these regions via kernel density modelling. Since there were no limma-significant probes in the treatment group comparison (FDR-adjusted *P* < 0.05) to search for DMRs, we relaxed the DMRcate default parameters that threshold significance based on the number of limma-significant probes. Parameterizations included a bandwidth size of 500 bp = 1 standard deviation of Gaussian kernel support, non-default manual settings for the call to DMRcate of *P* cut-off = 0.05 for VMRs and 0.001 for DMRs, and selection of regions consisting of at least two consecutive CpG sites. To test the likelihood of detecting false-positive DMRs, we compared the number of significant DMRs called with an FDR *P* value cut-off of <0.001 for the treatment group comparison to the number of DMRs that would be identified by chance, using 500 random permutations of the group variable for DMRcate.

The association of DNA methylation at birth with cord blood DHA concentration, independently of treatment group, was tested using linear regression, again via *limma*, both with and without adjustment for gestational age.

## Additional files

Additional file 1: Table S1. Global DNA methylation levels at birth across all probes by genomic location. **Table S2.** Variable methylated regions (VMRs) at birth. **Table S3.** DMRs between the DHA-supplemented and control group at birth with adjustment for cell mixture. **Table S4.** DMRs between the DHA-supplemented and control group at birth in males. **Table S5.** DMRs between the DHA-supplemented and control group at birth in females. **Table S6.** DMRs between the DHA-supplemented and control group at age 5 years. **Table S7.** DMRs between the DHA-supplemented and control group at age 5 years in males. **Table S8.** DMRs between the DHA-supplemented and control group at age 5 years in females. (XLSX 476 kb)
Additional file 2: Figure S1. Distribution of *limma t*-statistic and *limma* –log2(*P* value) for the comparison of the DHA and control group in all children (black) and males (blue) and females (pink) separately. **Figure S2.** Number of differentially methylated regions (DMRs) in permutation tests. The number of actual DMRs between the DHA and the control group (red line) in the whole population and males and females separately, compared to the number of DMRs from 500 random permutations of the group variable. (DOCX 183 kb)

## References

[1] Waterland RA, Michels KB (2007). Epigenetic epidemiology of the developmental origins hypothesis. Annu Rev Nutr.

[2] Barker DJ, Godfrey K, Gluckman P, Harding J, Owens J, Robinson J (1993). Fetal nutrition and cardiovascular disease in adult life. Lancet.

[3] Gluckman PD, Lillycrop KA, Vickers MH, Pleasants AB, Phillips ES, Beedle AS, Burdge GC, Hanson MA (2007). Metabolic plasticity during mammalian development is directionally dependent on early nutritional status. Proc Natl Acad Sci U S A.

[4] Williams L, Seki Y, Vuguin PM, Charron MJ (2014). Animal models of in utero exposure to a high fat diet: a review. Biochim Biophys Acta.

[5] Dominguez-Salas P, Moore SE, Baker MS, Bergen AW, Cox SE, Dyer RA, Fulford AJ, Guan Y, Laritsky E, Silver MJ, Swan GE, Zeisel SH, Innis SM, Waterland RA, Prentice AM, Hennig BJ (2014). Maternal nutrition at conception modulates DNA methylation of human metastable epialleles. Nat Commun.

[6] Tobi EW, Goeman JJ, Monajemi R, Gu H, Putter H, Zhang Y, Slieker RC, Stok AP, Thijssen PE, Müller F, van Zwet EW, Bock C, Meissner A, Lumey LH, Eline Slagboom P, Heijmans BT (2014). DNA methylation signatures link prenatal famine exposure to growth and metabolism. Nat Commun.

[7] Joubert BR, Håberg SE, Nilsen RM, Wang X, Vollset SE, Murphy SK, Huang Z, Hoyo C, Midttun Ø, Cupul-Uicab LA, Ueland PM, Wu MC, Nystad W, Bell DA, Peddada SD, London SJ (2012). 450K epigenome-wide scan identifies differential DNA methylation in newborns related to maternal smoking during pregnancy. Environ Health Perspect.

[8] Koestler DC, Avissar-Whiting M, Houseman EA, Karagas MR, Marsit CJ (2013). Differential DNA methylation in umbilical cord blood of infants exposed to low levels of arsenic in utero. Environ Health Perspect.

[9] Joubert BR, den Dekker HT, Felix JF, Bohlin J, Ligthart S, Beckett E, Tiemeier H, van Meurs JB, Uitterlinden AG, Hofman A, Haberg SE, Reese SE, Peters MJ, Kulle Andreassen B, Steegers EAP, Nilsen RM, Vollset SE, Midttun O, Ueland PM, Franco OH, Dehghan A, de Jongste JC, Wu MC, Wang T, Peddada SD, Jaddoe VWV, Nystad W, Duijts L, London SJ (2016). Maternal plasma folate impacts differential DNA methylation in an epigenome-wide meta-analysis of newborns. Nat Commun.

[10] Khulan B, Cooper WN, Skinner BM, Bauer J, Owens S, Prentice AM, Belteki G, Constancia M, Dunger D, Affara NA (2012). Periconceptional maternal micronutrient supplementation is associated with widespread gender related changes in the epigenome: a study of a unique resource in the Gambia. Hum Mol Genet.

[11] Cooper WN, Khulan B, Owens S, Elks CE, Seidel V, Prentice AM, Belteki G, Ong KK, Affara NA, Constância M, Dunger DB (2012). DNA methylation profiling at imprinted loci after periconceptional micronutrient supplementation in humans: results of a pilot randomized controlled trial. FASEB J.

[12] Guenard F, Deshaies Y, Cianflone K, Kral JG, Marceau P, Vohl M-C (2013). Differential methylation in glucoregulatory genes of offspring born before vs. after maternal gastrointestinal bypass surgery. Proc Natl Acad Sci.

[13] Lorente-Cebrián S, Costa AGV, Navas-Carretero S, Zabala M, Martínez JA, Moreno-Aliaga MJ (2013). Role of omega-3 fatty acids in obesity, metabolic syndrome, and cardiovascular diseases: a review of the evidence. J Physiol Biochem.

[14] Rogers LK, Valentine CJ, Keim SA (2013). DHA supplementation: current implications in pregnancy and childhood. Pharmacol Res.

[15] Larqué E, Gil-Sánchez A, Prieto-Sánchez MT, Koletzko B (2012). Omega 3 fatty acids, gestation and pregnancy outcomes. Br J Nutr.

[16] De Giuseppe R, Roggi C, Cena H (2014). n-3 LC-PUFA supplementation: effects on infant and maternal outcomes. Eur J Nutr.

[17] Hauner H, Brunner S, Amann-Gassner U (2013). The role of dietary fatty acids for early human adipose tissue growth. Am J Clin Nutr.

[18] Burdge GC, Lillycrop KA (2014). Fatty acids and epigenetics. Curr Opin Clin Nutr Metab Care.

[19] Kulkarni A, Dangat K, Kale A, Sable P, Chavan-Gautam P, Joshi S (2011). Effects of altered maternal folic acid, vitamin B12 and docosahexaenoic acid on placental global DNA methylation patterns in Wistar rats. PLoS One.

[20] Meher A, Joshi A, Joshi S (2014). Differential regulation of hepatic transcription factors in the Wistar rat offspring born to dams fed folic acid, vitamin B12 deficient diets and supplemented with omega-3 Fatty acids. PLoS One.

[21] Aslibekyan S, Wiener HW, Havel PJ, Stanhope KL, Brien DMOO, Hopkins SE, Absher DM, Tiwari HK, Boyer BB (2014). DNA methylation patterns are associated with n – 3 fatty acid intake in Yup Õ ik People 1 – 3. J Nutr.

[22] Voisin S, Almén MS, Moschonis G, Chrousos GP, Manios Y, Schiöth HB (2014). Dietary fat quality impacts genome-wide DNA methylation patterns in a cross-sectional study of Greek preadolescents. Eur J Hum Genet.

[23] Hoile SP, Clarke-Harris R, Huang R-C, Calder PC, Mori TA, Beilin LJ, Lillycrop KA, Burdge GC (2014). Supplementation with N-3 long-chain polyunsaturated fatty acids or olive oil in men and women with renal disease induces differential changes in the DNA methylation of FADS2 and ELOVL5 in peripheral blood mononuclear cells. PLoS One.

[24] Lee H-S, Barraza-Villarreal A, Hernandez-Vargas H, Sly PD, Biessy C, Ramakrishnan U, Romieu I, Herceg Z (2013). Modulation of DNA methylation states and infant immune system by dietary supplementation with ω-3 PUFA during pregnancy in an intervention study. Am J Clin Nutr.

[25] Lee H-S, Barraza-Villarreal A, Biessy C, Duarte-Salles T, Sly PD, Ramakrishnan U, Rivera J, Herceg Z, Romieu I (2014). Dietary supplementation with polyunsaturated fatty acid during pregnancy modulates DNA methylation at IGF2/H19 imprinted genes and growth of infants. Physiol Genomics.

[26] Amarasekera M, Noakes P, Strickland D, Saffery R, Martino DJ, Prescott SL (2014). Epigenome-wide analysis of neonatal CD4(+) T-cell DNA methylation sites potentially affected by maternal fish oil supplementation. Epigenetics.

[27] Feinberg AP, Irizarry RA, Fradin D, Aryee MJ, Murakami P, Aspelund T, Eiriksdottir G, Harris TB, Launer L, Gudnason V, Fallin MD (2010). Personalized epigenomic signatures that are stable over time and covary with body mass index. Sci Transl Med.

[28] Lemire M, Zaidi SHE, Ban M, Ge B, Aïssi D, Germain M, Kassam I, Wang M, Zanke BW, Gagnon F, Morange P-E, Trégouët D-A, Wells PS, Sawcer S, Gallinger S, Pastinen T, Hudson TJ (2015). Long-range epigenetic regulation is conferred by genetic variation located at thousands of independent loci. Nat Commun.

[29] Volkov P, Olsson AH, Gillberg L, Jørgensen SW, Brøns C, Eriksson KF, Groop L, Jansson PA, Nilsson E, Rönn T, Vaag A, Ling C (2016). A genome-wide mQTL analysis in human adipose tissue identifies genetic variants associated with DNA methylation, gene expression and metabolic traits. PLoS One.

[30] Teh AL, Pan H, Chen L, Ong M-L, Dogra S, Wong J, MacIsaac JL, Mah SM, McEwen LM, Saw S-M, Godfrey KM, Chong Y-S, Kwek K, Kwoh C-K, Soh S-E, Chong MFF, Barton S, Karnani N, Cheong CY, Buschdorf JP, Stünkel W, Kobor MS, Meaney MJ, Gluckman PD, Holbrook JD (2014). The effect of genotype and in utero environment on interindividual variation in neonate DNA methylomes. Genome Res.

[31] Lam LL, Emberly E, Fraser HB, Neumann SM, Chen E, Miller GE, Kobor MS (2012). Factors underlying variable DNA methylation in a human community cohort. Proc Natl Acad Sci U S A.

[32] Fernández-Busnadiego R, Saheki Y, De Camilli P (2015). Three-dimensional architecture of extended synaptotagmin-mediated endoplasmic reticulum-plasma membrane contact sites. Proc Natl Acad Sci U S A.

[33] Kahle KT, Staley KJ, Nahed BV, Gamba G, Hebert SC, Lifton RP, Mount DB (2008). Roles of the cation-chloride cotransporters in neurological disease. Nat Clin Pract Neurol.

[34] Lieverse RJ, Jansen JBMJ, Masclee AAM, Lamers CBHW (1994). Role of cholecystokinin in the regulation of satiation and satiety in humans. Ann N Y Acad Sci.

[35] Lanier LL (2015). NKG2D receptor and its ligands in host defense. Cancer Immunol Res.

[36] Agyekum S, Church A, Sohail M, Krausz T, Van Noorden S, Polak J, Cohen J (2003). Expression of lymphotoxin-beta (LT-beta) in chronic inflammatory conditions. J Pathol.

[37] Arcos-Burgos M, Jain M, Acosta MT, Shively S, Stanescu H, Wallis D, Domené S, Vélez JI, Karkera JD, Balog J, Berg K, Kleta R, Gahl WA, Roessler E, Long R, Lie J, Pineda D, Londoño AC, Palacio JD, Arbelaez A, Lopera F, Elia J, Hakonarson H, Johansson S, Knappskog PM, Haavik J, Ribases M, Cormand B, Bayes M, Casas M, Ramos-Quiroga JA, Hervas A, Maher BS, Faraone SV, Seitz C, Freitag CM, Palmason H, Meyer J, Romanos M, Walitza S, Hemminger U, Warnke A, Romanos J, Renner T, Jacob C, Lesch K-P, Swanson J, Vortmeyer A, Bailey-Wilson JE, Castellanos FX, Muenke M (2010). A common variant of the latrophilin 3 gene, LPHN3, confers susceptibility to ADHD and predicts effectiveness of stimulant medication. Mol Psychiatry.

[38] Bonnefont J, Nikolaev SI, Perrier AL, Guo S, Cartier L, Sorce S, Laforge T, Aubry L, Khaitovich P, Peschanski M, Antonarakis SE, Krause K-H (2008). Evolutionary forces shape the human RFPL1,2,3 genes toward a role in neocortex development. Am J Hum Genet.

[39] Simpkin AJ, Suderman M, Gaunt TR, Lyttleton O, McArdle WL, Ring SM, Tilling K, Davey Smith G, Relton CL (2015). Longitudinal analysis of DNA methylation associated with birth weight and gestational age. Hum Mol Genet.

[40] Makrides M, Gibson RA, McPhee AJ, Yelland L, Quinlivan J, Ryan P (2010). Effect of DHA supplementation during pregnancy on maternal depression and neurodevelopment of young children: a randomized controlled trial. JAMA.

[41] Aiken CE, Ozanne SE (2012). Sex differences in developmental programming models. Reproduction.

[42] Tobi EW, Lumey LH, Talens RP, Kremer D, Putter H, Stein AD, Slagboom PE, Heijmans BT (2009). DNA methylation differences after exposure to prenatal famine are common and timing- and sex-specific. Hum Mol Genet.

[43] Cheong JN, Wlodek ME, Moritz KM, Cuffe JS (2016). Programming of maternal and offspring disease: impact of growth restriction, fetal sex and transmission across generations. J Physiol.

[44] Gaunt TR, Shihab HA, Hemani G, Min JL, Woodward G, Lyttleton O, Zheng J, Duggirala A, McArdle WL, Ho K, Ring SM, Evans DM, Davey Smith G, Relton CL (2016). Systematic identification of genetic influences on methylation across the human life course. Genome Biol.

[45] Richmond RC, Simpkin AJ, Woodward G, Gaunt TR, Lyttleton O, McArdle WL, Ring SM, Smith ADAC, Timpson NJ, Tilling K, Davey Smith G, Relton CL (2015). Prenatal exposure to maternal smoking and offspring DNA methylation across the lifecourse: findings from the Avon Longitudinal Study of Parents and Children (ALSPAC). Hum Mol Genet.

[46] Bakulski KM, Feinberg JI, Andrews SV, Yang J, Brown S, McKenney S, Witter F, Walston J, Feinberg AP, Fallin MD (2016). DNA methylation of cord blood cell types: applications for mixed cell birth studies. Epigenetics.

[47] Makrides M, Gould JF, Gawlik NR, Yelland LN, Smithers LG, Anderson PJ, Gibson RA (2014). Four-year follow-up of children born to women in a randomized trial of prenatal DHA supplementation. JAMA.

[48] Muhlhausler BS, Yelland LN, McDermott R, Tapsell L, McPhee AJ, Gibson RA, Makrides M (2016). DHA supplementation during pregnancy does not reduce BMI or body fat mass in children: follow-up of the DOMInO randomized controlled trial. Am J Clin Nutr.

[49] Palmer DJ, Sullivan T, Gold MS, Prescott SL, Heddle R, Gibson RA, Makrides M (2012). Effect of n-3 long chain polyunsaturated fatty acid supplementation in pregnancy on infants’ allergies in first year of life: randomised controlled trial. BMJ.

[50] van Dijk SJ, Molloy PL, Varinli H, Morrison JL, Muhlhausler BS (2014). Epigenetics and human obesity. Int J Obes (Lond).

[51] Rand KN, Molloy PL (2010). Sensitive measurement of unmethylated repeat DNA sequences by end-specific PCR. Biotechniques.

[52] Pidsley R, Wong CCY, Volta M, Lunnon K, Mill J, Schalkwyk LC (2013). A data-driven approach to preprocessing Illumina 450K methylation array data. BMC Genomics.

[53] Chen Y, Lemire M, Choufani S, Butcher DT, Grafodatskaya D, Zanke BW, Gallinger S, Hudson TJ, Weksberg R (2013). Discovery of cross-reactive probes and polymorphic CpGs in the Illumina Infinium HumanMethylation450 microarray. Epigenetics.

[54] Oytam Y, Sobhanmanesh F, Duesing K, Bowden JC, Osmond-McLeod M, Ross J (2016). Risk-conscious correction of batch effects: maximising information extraction from high-throughput genomic datasets. BMC Bioinformatics.

[55] Ritchie ME, Phipson B, Wu D, Hu Y, Law CW, Shi W, Smyth GK (2015). limma powers differential expression analyses for RNA-sequencing and microarray studies. Nucleic Acids Res.

[56] Benjamini Y, Hochberg Y (1995). Controlling the false discovery rate: a practical and powerful approach to multiple testing on JSTOR. J R Stat Soc.

[57] Peters TJ, Buckley MJ, Statham AL, Pidsley R, Samaras K, Lord RV, Clark SJ, Molloy PL (2015). De novo identification of differentially methylated regions in the human genome. Epigenetics Chromatin.

