# Supplementary material for: Effect of prenatal DHA supplementation on the infant epigenome: results from a randomized controlled trial
Source: Clin Epigenetics. 2016 Nov 4;8:114. doi: 10.1186/s13148-016-0281-7 (PMC5096291; doi:10.1186/s13148-016-0281-7)
Supplement: Additional file 2: Figure S1. — Distribution of limma t-statistic and limma –log2(P value) for the comparison of the DHA and control group in all children (black) and males (blue) and females (pink) separately. Figure S2. Number of differentially methylated regions (DMRs) in permutation tests. The number of actual DMRs between the DHA and the control group (red line) in the whole population and males and females separately, compared to the number of DMRs from 500 random permutations of the group variable. (DOCX 183 kb) [file 13148_2016_281_MOESM2_ESM.docx]

**SUPPLEMENTAL FIGURES**


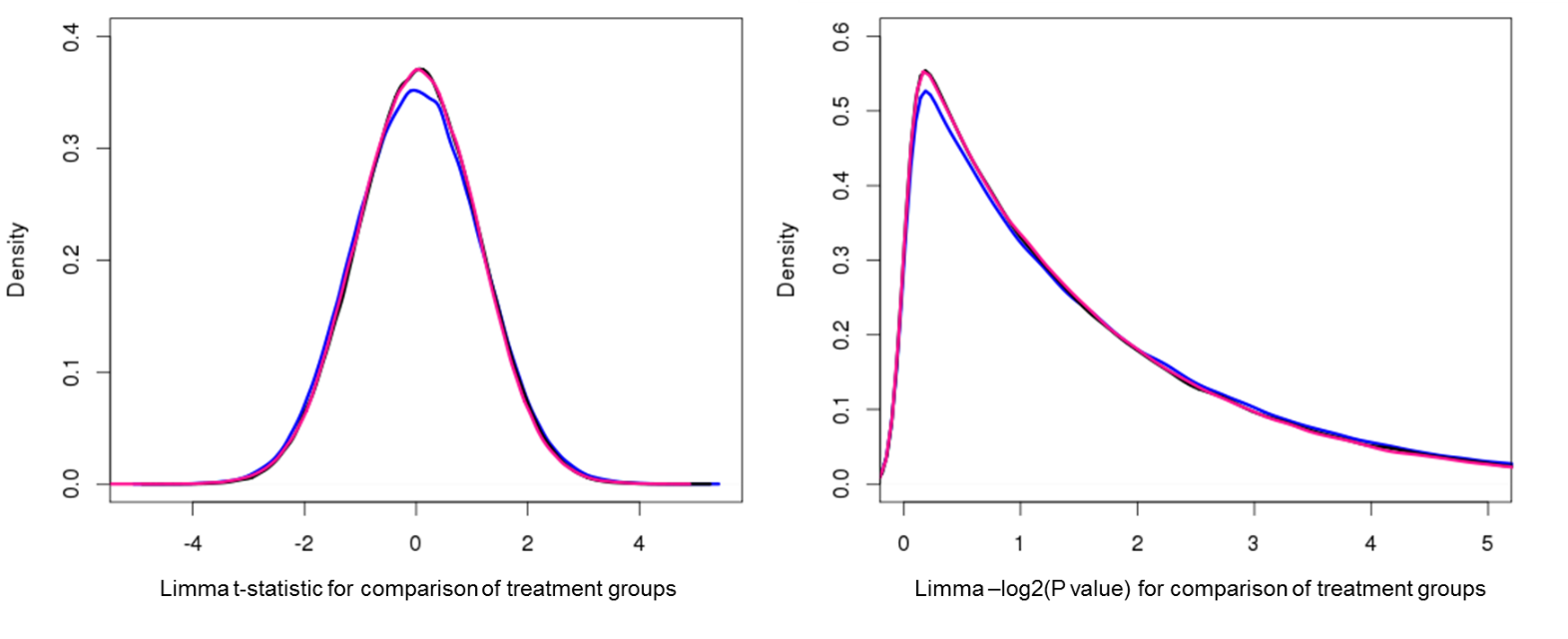


**Figure S1** Distribution of *limma* t-statistic and *limma* –log2(P value) for the comparison of the DHA and control group in all children (black) and males (blue) and females (pink) separately


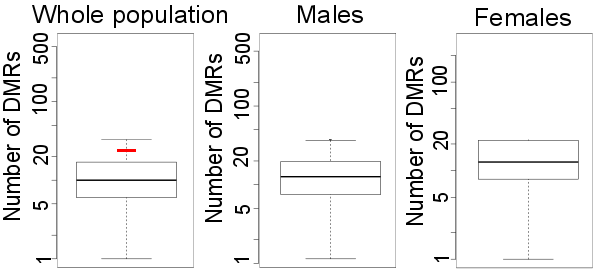


**Figure S2** Number of differentially methylated regions (DMRs) in permutation tests. The number of actual DMRs between the DHA and the control group (red line) in the whole population and males and females separately, compared to the number of DMRs from 500 random permutations of the group variable.
